# A threatened new species of *Ipomoea* (Convolvulaceae) from the Brazilian Cerrado revealed by morpho-anatomical analysis

**DOI:** 10.3897/phytokeys.151.49833

**Published:** 2020-06-17

**Authors:** Diego Santos, Raysa Valéria Carvalho Saraiva, Tiago Massi Ferraz, Emília Cristina Pereira Arruda, Maria Teresa Buril

**Affiliations:** 1 Programa de Pós-Graduação em Botânica, Laboratório de Sistemática Integrativa, Universidade Federal Rural de Pernambuco, Av. Manoel de Medeiros, s/n, Dois Irmãos, 50670-901, Recife, Pernambuco, Brazil Universidade Federal Rural de Pernambuco Recife Brazil; 2 Programa de Pós-Graduação em Agroecologia, Universidade Estadual do Maranhão, Av. Lourenço Vieira da Silva, s/n, Jardim São Cristóvão, 65055-970, São Luís, Maranhão, Brazil Universidade Estadual do Maranhão São Luís Brazil; 3 Departamento de Botânica, Laboratório de Anatomia Vegetal, Universidade Federal de Pernambuco, Av. Prof. Moraes Rego, 1235, Cidade Universitária, 50670-901, Recife, Pernambuco, Brazil Universidade Federal de Pernambuco Recife Brazil

**Keywords:** biodiversity, Brazilian flora, conservation, endangered species, endemic species, morpho-anatomy, South America

## Abstract

A new species of *Ipomoea*, endemic to the Cerrado domain in Maranhão, Brazil, is described. *Ipomoea maranhensis* D.Santos & Buril, **sp. nov.** has been misidentified as *I. burchellii* Meisn. in several herbaria. Even though both species have oblong, pubescent leaves, they can be distinguished by morpho-anatomical characters. We present a diagnosis, complete description, illustration, taxonomic comments, conservation status and distribution map.

## Introduction

*Ipomoea* (Convolvulaceae) comprises approximately 700 species (Staples 2015) and is widely distributed throughout the world, but is predominantly pantropical and absent in Mediterranean areas and temperate climates (Staples and Brummitt 2007). In Brazil, it is represented by 149 species distributed in all regions and phytogeographic domains (Flora do Brasil 2019). The genus can be distinguished by its echinate pollen (Simão-Bianchini 1998). Recently, knowledge of *Ipomoea* has been increased with the publication of several new species (e.g. Wood et al. 2015; Vasconcelos et al. 2016; Wood et al. 2017a, b; Wood and Scotland 2017a, b), many of which are endemic to Brazil (Wood et al. 2017c; Santos et al. 2019; Santos et al. in press).

Among Brazilian phytogeographic domains, the Cerrado stands out as a center of diversity and endemism for several plant groups (Simon et al. 2009). Despite being one of the 34 global biodiversity hotspots (Mittermeier et al. 2004), this domain has been intensely devastated in recent decades due to the expansion of agriculture and livestock (Cunha et al. 2008). Of the 12,113 Cerrado angiosperm species (BFG 2018), 366 are considered endangered (Martinelli et al. 2014). The genus *Ipomoea* is represented by 92 species in the Cerrado biome, which corresponds to 22% of the family’s diversity in the country (Flora do Brasil 2019). Of these, *I. macedoi* Hoehne, *I. maurandioides* Meisn. and *I. sobrevoluta* Choisy are considered endangered and do not occur in protected areas (Martinelli and Moraes 2013), and may become extinct if we do not take any conservation initiative.

While analyzing *Ipomoea* collections from CEN, HST, HUEFS, and SLUI (acronyms follow Thiers 2019), we found a morphotype collected in the municipality of Carolina, Chapada das Mesas National Park in the State of Maranhão, that was dubiously identified as *I. burchellii* Meisn. When we analyzed the protologue and the type collection of *I. burchellii*, we noted significant differences in the morphology and indumentum of sepals between this species and the morphotype. After analyzing several *I. burchellii* specimens, we found that these differences are consistent.

We consulted literature related to climbing and subshrubby species of *Ipomoea* from South America (O’Donell 1948; O’Donell 1959a, b; Simão-Bianchini 1998; Léon 2006; Krapovickas 2009; Wood et al. 2015; Wood et al. 2017a, b, c; Wood and Scotland 2017a, b), type specimens from online database (http://plants.jstor.org), and Brazilian herbaria through *SpeciesLink* network (http://www.splink.org.br) in order to investigate similiar species to this morphotype. However, the combination of morphological features found in the morphotype did not match those of any known species.

To support the morphological delimitation between this morphotype and *I. burchellii*, we performed a comparative anatomical analysis of the leaf, as this has been used to support the morphological delimitation of species in various plant groups (Lersten 1974; Gomes et al. 2005; Rio et al. 2005; Oliveira et al. 2011; Thadeo et al. 2014), as well as in Convolvulaceae (Ketjarun et al. 2016; Traiperm et al. 2017). Thus, considering the taxonomic alleged consistency of the anatomical characters analyzed in Convolvulaceae (Metcalfe and Chalk 1979), along with the morphological discontinuities found, we describe this morphotype as a new species.

## Methods

### Morphological analysis

We analyzed specimens from the following herbaria: CEN, HUEFS, SLUI (acronyms follow Thiers 2019), and HST (unindexed herbarium from the Universidade Federal Rural de Pernambuco). Morphological terminology followed Harris and Harris (1994). The specimen collected in the Carolina municipality, Chapada das Mesas National Park, Maranhão state, was preserved according to standard taxonomic techniques (Mori et al. 1989) and deposited in SLUI. The main diagnostic characters of the species were illustrated based on the type specimen.

### Comparative anatomical analysis of the leaf

For comparative anatomical analyses, three *I. burchellii* specimens and two vouchers of the new species were included in this study (Table 1). Three leaves from the fourth node and parts of the petiole of each specimen were rehydrated according to Smith and Smith (1942). Then, the samples were placed in 2% potassium hydroxide solution at room temperature for two hours. Subsequently, the material was washed with distilled water three times. The samples were progressively dehydrated from 10% alcohol until their final storage in 70% ethanol (Johansen 1940). The median region of blade and petioles were free-hand sectioned and clarified with 50% sodium hypochlorite and stained with safrablue (safranin and Astra-Blau, Bukatsch 1972). The slides were prepared with glycerin and fixed with enamel (Kraus and Arduin 1997). The slides were deposited into the Plant Anatomy Laboratory (LAVeg) at the Universidade Federal de Pernambuco. The analysis and documentation were performed under a Leica DM500 microscope. The anatomical terminology followed Metcalfe and Chalk (1979).

**Table 1.** List of vouchers sampled for comparative anatomical analysis of *Ipomoea maranhensis* and *I. burchellii*.

Species/specimens	Collection point	Voucher	Herbarium
*I. maranhensis*			
specimen 1	Ibipira, Mirador, Maranhão state	L.P. Felix et al. 8136	HST
specimen 2	Carolina, Maranhão state	R.V.C. Saraiva 107	SLUI
*I. burchellii*			
specimen 1	Canápolis, Bahia state	Yoshida-Arns, K. 557	HUEFS
specimen 2	São Desidério, Bahia state	A.M. Miranda 3787	HST
specimen 3	Paraiso, Goiás state	Irwin, H.S. 21745	HUEFS

### Conservation status

Distribution records were obtained from herbarium sheets. The conservation status was based on IUCN guidelines and criteria (IUCN 2019) using georeferenced data from cited collections. The area of occupancy (AOO) and extent of occurrence (EOO) were calculated using GeoCAT (Bachman et al. 2011). The distribution map was created using the QGIS version 2.7 software (QGIS Development Team 2015).

## Taxonomic treatment

### Ipomoea maranhensis

Taxon classificationPlantaeSolanalesConvolvulaceae

D. Santos & Buril sp. nov.

0A828ABD-A6B9-5E9E-B154-0A6283A26D3D

urn:lsid:ipni.org:names:77209927-1

Figs 1 , 2

#### Type.

Brazil. Maranhão: município de Mirador, Ibipira, Parque Estadual do Mirador, 06°22'01"S, 44°22'00"W, 11 April 1998 (fl.), *L.P. Félix et al. 8136* (holotype HUEFS38133, isotype HST8028).

#### Diagnosis.

*Ipomoea maranhensis* differs morphologically from *I. burchellii* Meins. by its twining habit (vs. subshrubs), lanceolate (vs. oblong), long-acuminate (vs. acute) and densely sericeous (vs. hirsute) sepals.

**Figure 1.**
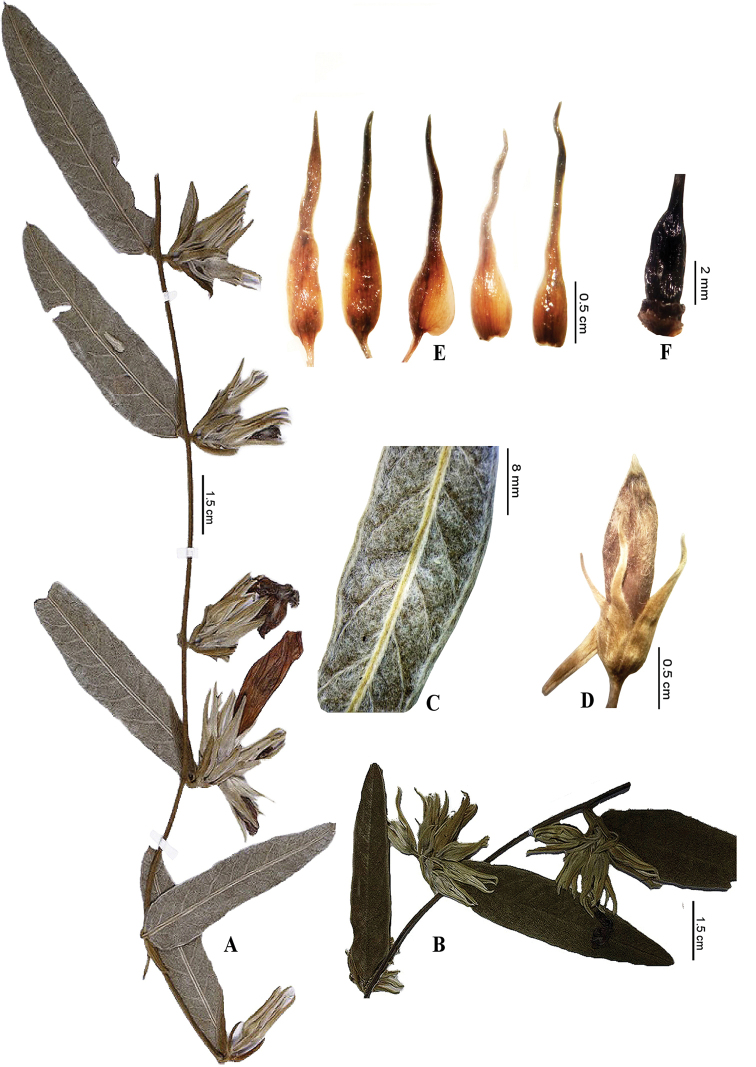
**A–F***Ipomoea maranhensis***A** branch with leaves (abaxial surface) and flowers **B** branch with leaves (adaxial surface) and floral buds **C** leaf in abaxial view presenting sericeous aspect **D** floral bud **E** sepals **F** ovary (Photos by F. Santos and Flora do Brasil 2019).

#### Description.

Vine, internodes 1–5 cm long, latex absent; stem pubescent with whitish trichomes. Leaf blade 3.3–7 × 0.9–2.3 cm, oblong, base cuneate, rounded to subcordate, apex obtuse, acute, apiculate, margin entire, pubescent on both surfaces, discolor, whitish on abaxial face, brochidodromous, 6–10 pairs of secondary veins, main vein prominent; petiole 2–3 mm long, canaliculate, pubescent, nectary near the apex of the petiole. Inflorescence of dichasial cymes reduced, bracteolate in the axils of the upper leaves, with 3–7 flowers; peduncle 2–3 mm long, pubescent; bracts 1.7–2 × 0.5–0.8 cm, elliptical, base cuneate, apex obtuse, pubescent, foliaceous, discolor; pedicel 2–4 mm long, pubescent; bracteoles 0.9–1.6 cm long, lanceolate, oblong, base truncate, apex acute, obtuse, margin entire, pubescent. Sepals all equal, 1.7–2.3 × 0.4–0.5 cm, lanceolate, base cuneate, apex long-acuminate, margins non-membranous, densely sericeous on the adaxial surface of the sepals, whitish. Corolla ca. 4 cm long, ca. 4.5 cm diam, infundibuliform, tube ca. 1.6 cm long, rose with whitish tube, midpetaline bands area sericeous externally. Stamens ca. 3 cm long, included, unequal, pilose at the base, anthers ca. 4 mm long, oblong, white. Ovary ca. 4 mm long, conical, glabrous, 4 locule; style ca. 2 cm long, glabrous; stigma bi-globose, papillose. Fruit unknown.

**Figure 2.**
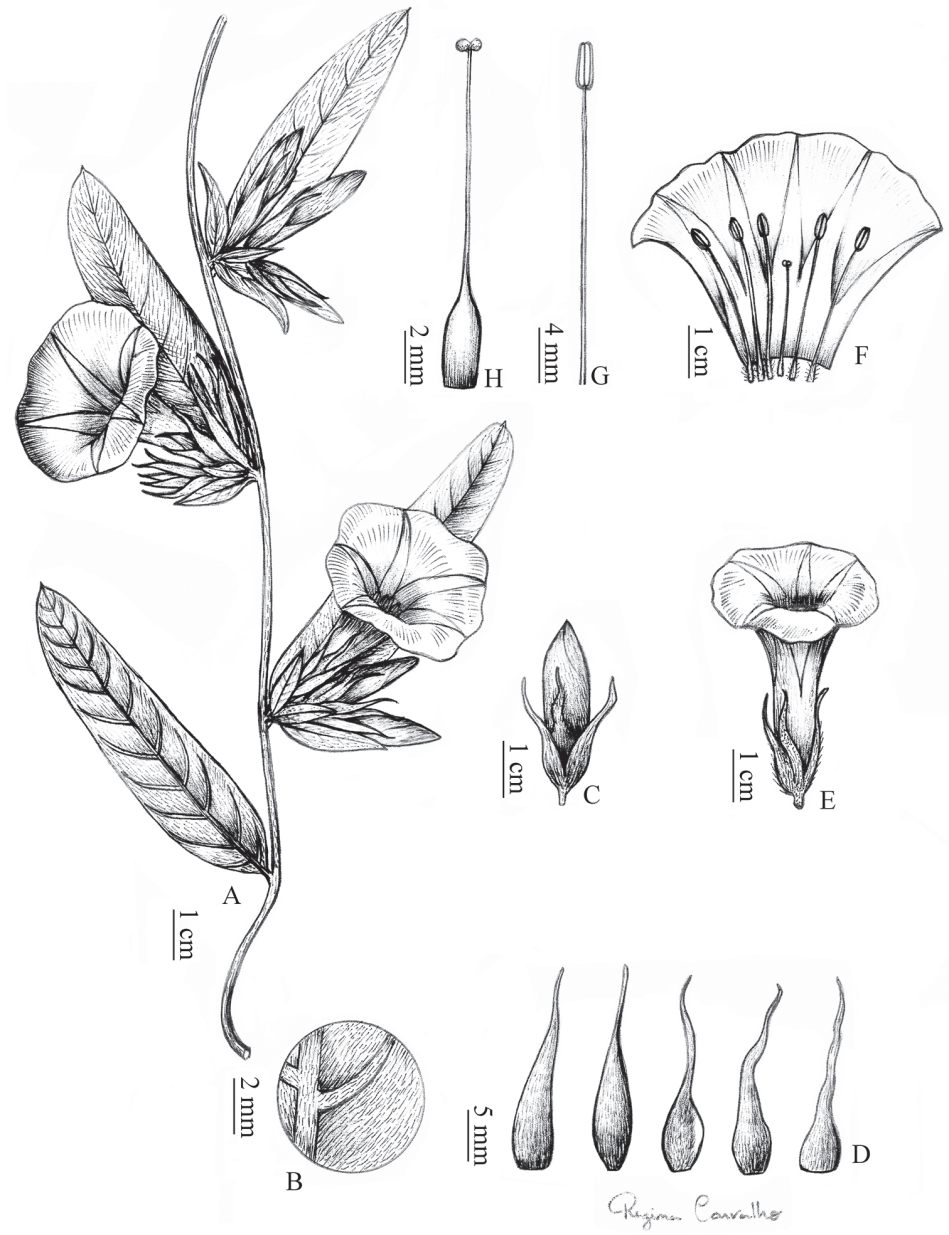
**A–H***Ipomoea maranhensis***A** twining habit **B** primary and secondary veins on the abaxial surface **C** floral bud **D** sepals with apex long acuminate **E** flower **F** open corolla **G** stamen **H** gynoecium. Drawn by Regina Carvalho from *Félix et al. 8136*.

#### Anatomical description – petiole and leaf blade.

Petiole epidermis uniseriate with juxtaposed cells, parenchyma with isodiametric cells, prominent/concave main rib shape, U-shaped vascular bundles. Leaf epidermis uniseriate, mesophyll dorsiventral with crystallized idioblasts (druses), palisade parenchyma with one to two layers of elongated cells more evident on the adaxial surface, spongy parenchyma with three to four layers of round cells with sinuous anticlinal walls; glandular trichomes on both leaf surfaces.

**Figure 3.**
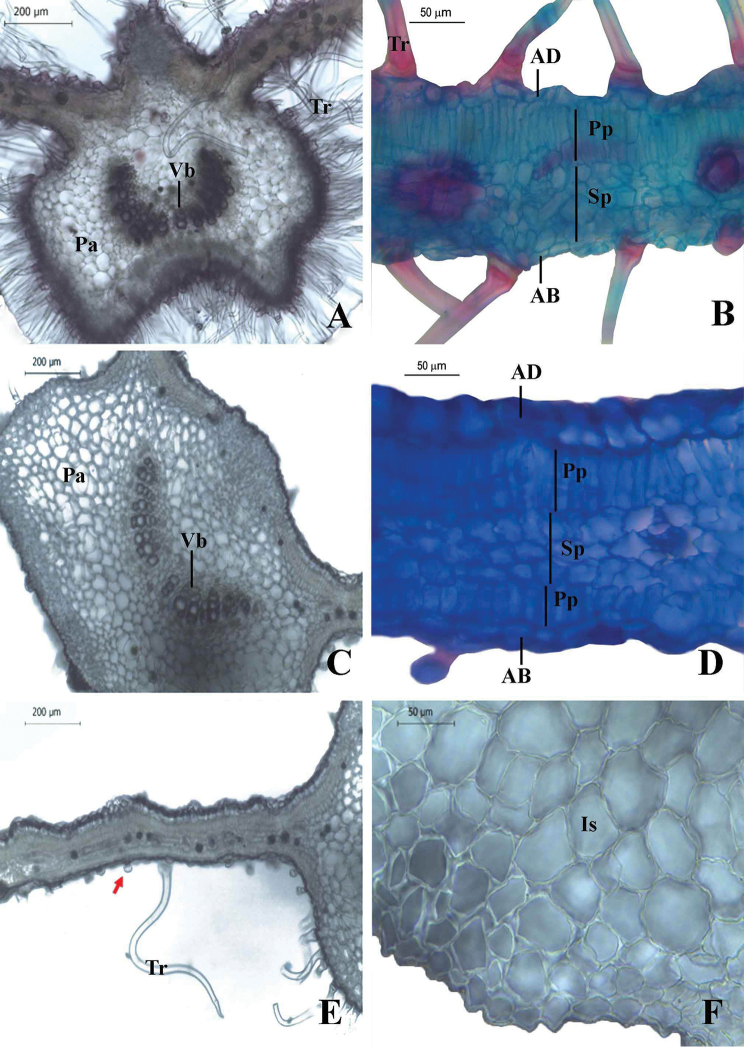
**A, B***Ipomoea maranhensis***A** arrangement of vascular bundles in U; prominent/concave main rib shape **B** dorsiventral mesophyll **C–F***I. burchellii***C** arrangement of vascular bundles in V; convex/flat main rib shape **D** mesophyll isobilateral **E** glandular trichomes restricted to the abaxial surface of the leaf (red arrow) **F** parenchymatous tissue with isodiametric cells (Photos by E. Pereira Arruda). AD: adaxial epidermis; AB: abaxial epidermis; Is: isodiametric cells; Pa: Parenchymatous tissue; Pp: palisade parenchyma; Sp: spongy parenchyma; Vb: vascular bundle; Tr: trichome.

According to the anatomical analysis, we observed that *I. maranhensis* and *I. burchellii* share mesophyll with crystallized idioblasts (druses) and petiole parenchyma with isodiametric cells. However, *I. maranhensis* is distinguished from *I. burchellii* by a prominent/concave main rib shape (vs. convex/flat main rib shape in *I. burchellii*), U-shaped vascular bundles (vs. V-shaped), glandular trichomes spread on both leaf surfaces (vs. only on abaxial one) and dorsiventral mesophyll (vs. isobilateral) (Fig. 3) (Table 2).

**Table 2.** Comparison of morpho-anatomical characters of *Ipomoea maranhensis* and *I. burchellii*.

Characters	*I. maranhensis*	*I. burchellii*
Shape of sepals	Lanceolate	Oblong
Apex of sepals	Acuminate	Acute
Indumentum of sepals	Sericeous	Hirsute
Arrangement of vascular bundles	U-shaped	V-shaped
Glandular trichomes on leaf	Both surfaces	Abaxial surface
Type of mesophyll	Dorsiventral	Isobilateral

#### Phenology.

Collected with flowers in April.

#### Distribution and habitat.

**Figure 4.**
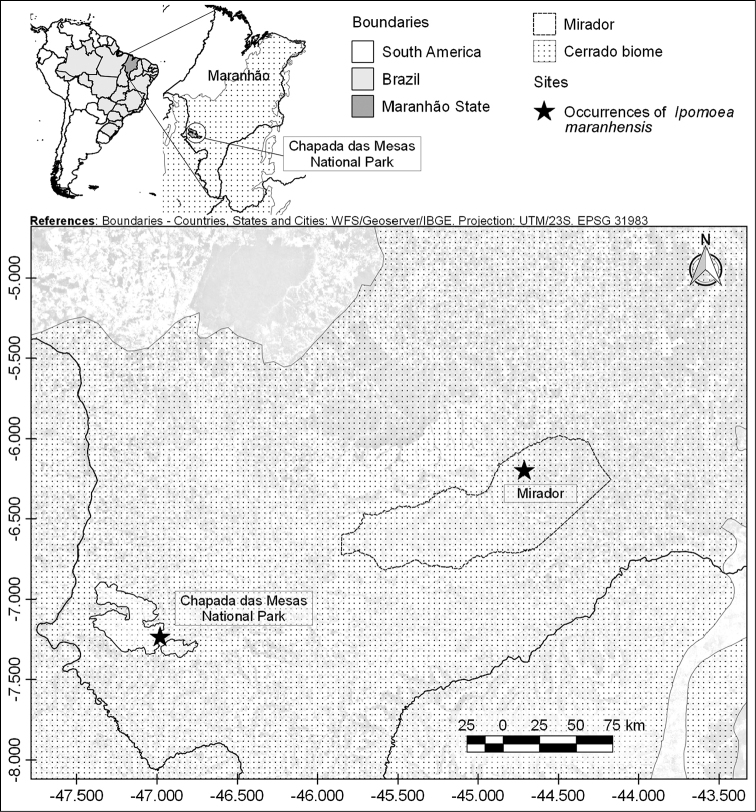
Distribution map of *Ipomoea maranhensis*.

*Ipomoea maranhensis* is known only from two disjunct populations between the municipality of Mirador, in the Mirador State Park (area of 4370 km^2^) and the municipality of Carolina, in the National Park Chapada das Mesas (CMNP, area of 1600 km^2^) (Fig. 4). In both areas this species grows on quartzite soils associated with Cerrado vegetation at 186–345 m elevation, average temperature of 26 °C and annual precipitation between 1250–1500 mm (Alcântara 2004; IBAMA 2013). The occurrence of this new species in the National Park Chapada das Mesas and in the Mirador State Park emphasizes the importance of these protected areas for the preservation of this taxon in the Cerrado domain.

#### Etymology.

The specific epithet refers to Maranhão state, where the type specimens were collected.

#### Additional specimens examined.

Brazil. Maranhão: Carolina, Parque Nacional da Chapada das Mesas, 345 m elev., 7°14'14"S, 46°58'50"W, 07 April 2017 (fl.), *R.V.C. Saraiva 107* (SLUI 5037); Parque Nacional Chapada das Mesas, accesso no Km 596 da BR – 230, 7 km E em estrada vicinal, 290 m elev., 7°07'33"S, 47°22'13"W, 12 April 2016 (fl.), *M.F. Simon et al. 2921* (CEN 95523).

#### Conservation status.

We categorized this species as Endangered (criteria EN) according to B1 (EOO < 5000 km^2^) and B2ab (ii, iii) (AOO < 500 km^2^) from IUCN (2019). Although populations of *I. maranhensis* occur in protected areas, this species is threatened due to the reduction of its habitat because of anthropogenic fires lit by small communities who remove vegetation with fire for subsistence agriculture in the Cerrado *sensu stricto* and *Cerradão* formations (Ribeiro and Walter 2008). According to Estivalet (1997), prostrate or climbing plants seem to be more prone to burning than upright species, whose growth points are more protected inside the clump.

### Identification key for *Ipomoea* species from the Chapada das Mesas National Park and Mirador State Park

**Table:** 

1	Leaf blade oblong, oblanceolate, linear or obovate	**2**
–	Leaf cordate, hastate, lobed	**5**
2	Leaf linear; sepals obovate, elliptic, glabrous	***I. schomburgkii* Meisn.**
–	Leaf oblong, oblanceolate, obovate; sepals oblong, ovate, lanceolate, sericeous, hirsute	**3**
3	Leaf oblanceolate to obovate; sepals ovate	***I. cuneifolia* A. Gray**
–	Leaf oblong; sepals oblong or lanceolate	**4**
4	Sepals oblong, obtuse, hirsute	***I. burchellii* Meisn.**
–	Sepals lanceolate, long-acuminate, sericeous	***I. maranhensis***
5	Sepals with subapical rostrum; corolla hypocrateriform	***I. hederifolia* L.**
–	Sepals lacking subapical rostrum; corolla funnelform	**6**
6	Leaf 5-lobed	***I. mauritiana* Jacq.**
–	Leaf hastate or cordate	**7**
7	Outer sepals unequal in size	***I. maurandioides* Meisn.**
–	Outer sepals equal in size	**8**
8	Sepals convex	***I. goyazensis* Gardner**
–	Sepals flat	***I. squamosa* Choisy**


## Discussion

The new species has been confused with *I. burchellii* because they share oblong, discolorous and sericeous leaves, flowers arranged in a dichasium and peduncle 2–3 mm long. However, according to the analysis of the type specimens (*Burchell 8738* deposited in K [K000612855]) and protologue of *I. burchellii*, the new species is morphologically different from *I. burchellii* by its habit, shape, apex, and indumentum of sepals. Misidentifications probably occurred because both species have oblong, discolorous leaves, 2–3 mm long peduncles and inflorescence arranged in dichasium, as well as because they occur in the Cerrado domain. Anatomical analysis revealed that the morphological delimitation of these species is supported by the shape of the vascular bundles, distribution of glandular trichomes in the leaf and type of mesophyll.

These characters are considered consistent (Metcalfe and Chalk 1979) and useful for Convolvulaceae taxonomy (e.g. Ketjarun et al. 2016; Traiperm et al. 2017). *Ipomoea maranhensis* can also be compared to *I. langsdorffii* Choisy, an endemic species from the Southeastern region of Brazil occuring in the Cerrado and Atlantic forest, due to its oblong leaves and flowers in dichasia. However, *I. maranhensis* can be distinguished from this species by its acute leaves (vs. obtuse in *I. langsdorffii*), lanceolate (vs. ovate), long-acuminate (vs. acute), and densely sericeous (vs. hirsute) sepals. These morphological characters present great taxonomic value for *Ipomoea* (Simão-Bianchini 1998; Ferreira and Miotto 2009; Wood et al. 2015).

One of the barriers that can hinder access to knowledge about the diversity of the genus *Ipomoea* is that several of its new species have been described based on the morphology from one or two specimens without the without using a tool to support the morphological delimitation (Wood et al. 2017a, b). Such a limited number of specimens can lead to misinterpretation about the consistency of characters, making it difficult to recognize these species. In these cases, investigating other sources of characters is important for preventing the proliferation of names that cause taxonomic confusion and nomenclatural instability. Anatomical studies have been used to support the morphological delimitation of species in various plant groups (Smith and Smith 1942; Lersten 1974; Gomes et al. 2005; Rio et al. 2005). Among the leaf anatomical characters used to support such delimitation are mesophyll type, main vein shape, and vascular bundle type (Gomes et al. 2005; Gomes et al. 2008; Zini et al. 2016).

In Convolvulaceae, these characters have also been consistent and informative, such as the type of mesophyll that was used to delimit three species of *Evolvulus* (Ketjarun et al. 2016), the shape of the main vein and vascular bundles used to clarify the relationship between morphologically similar *Argyrea* species (Traiperm et al. 2017). Furthermore, anatomical characters strongly supported species identification in an investigation of Merremia section Xanthips (Pisuttimarn et al. 2013). Such anatomical information has been used in these studies because it has proven to be useful and informative for taxonomic identification of plants (Thadeo et al. 2014).

## Supplementary Material

XML Treatment for Ipomoea maranhensis
